# Molecular genetic analysis and growth hormone response in patients with syndromic short stature

**DOI:** 10.1186/s12920-021-01113-8

**Published:** 2021-11-05

**Authors:** Huihui Sun, Na Li, Naijun Wan

**Affiliations:** 1 Department of Paediatrics, Beijing Jishuitan Hospital, No. 31 of Xinjiekou Dongjie Street, Xi Cheng District, Beijing, 100035 People’s Republic of China; 2 Department of Radiology, Beijing Jishuitan Hospital, No. 31 of Xinjiekou Dongjie Street, Xi Cheng District, Beijing, 100035 People’s Republic of China

**Keywords:** Syndromic short stature, Trio-whole-exome sequencing, Whole-genome low-coverage sequencing, *FGFR3*, *GNAS*, *TRPS1*

## Abstract

**Background:**

Syndromic short stature is a genetic and phenotypic heterogeneous disorder with multiple causes. This study aims to identify genetic causes in patients with syndromic short stature of unknown cause and evaluate the efficacy of the growth hormone response.

**Methods:**

Trio-whole-exome sequencing was applied to identify pathogenic gene mutations in seven patents with short stature, multiple malformations, and/or intellectual disability. Whole-genome low-coverage sequencing was also performed to identify copy number variants in three patients with concurrent intellectual disability. Recombinant human growth hormone was administered to improve height in patients with an identified cause of syndromic short stature.

**Results:**

Of the seven patients, three pathogenic/likely pathogenic gene mutations, including one *FGFR3* mutation (c.1620C>A p.N540K), one novel *GNAS* mutation (c.2288C>T p.A763V), and one novel *TRPS1* mutation (c.2527_c.2528dupTA p.S843fsX72), were identified in three patients. No copy number variants were identified in the three patients with concurrent intellectual disability. The proband with an *FGFR3* mutation, a female 4 and 3/12 years of age, was diagnosed with hypochondroplasia. Long-acting growth hormone improved her height from 85.8 cm [− 5.05 standard deviation (SD)] to 100.4 cm (− 4.02 SD), and her increased height SD score (SDS) was 1.03 after 25 months of treatment. The proband with a *GNAS* mutation, a female 12 and 9/12 years of age, was diagnosed with pseudohypoparathyroidism Ia. After 14 months of treatment with short-acting growth hormone, her height improved from 139.3 cm (− 2.69 SD) to 145.0 cm (− 2.36 SD), and her increased height SDS was 0.33.

**Conclusions:**

Trio-whole-exome sequencing was an important approach to confirm genetic disorders in patients with syndromic short stature of unknown etiology. Short-term growth hormone was effective in improving height in patients with hypochondroplasia and pseudohypoparathyroidism Ia.

## Background

Syndromic short stature is a phenotypic and genetically heterogeneous disease with a complex aetiology, including chromosomal aberrations, copy number variants (CNVs), gene mutations, methylation defects and other unknown causes [1]. Given the diversity and rarity of these disorders, precision diagnosis is difficult based on clinical manifestations and laboratory and radiological examination findings [2]. Molecular genetic analysis plays an important role in the precision diagnosis, proper treatment, prognosis, and genetic counselling of patients with short stature of unknown aetiology [3]. Recombinant human growth hormone (rhGH) use has been advocated in patients with Turner syndrome, Prader–Willi syndrome, and Noonan syndrome [4–6]. However, rhGH use is not routine in patients with many other syndromic short statures.

In our study, trio-whole exome sequencing (Trio-WES) was used to identify pathogenic gene mutations in patients with syndromic short stature of unknown aetiology [7]. Whole-genome low-coverage sequencing was also carried out to identify CNVs in patients with syndromic short stature combined with intellectual disability [8, 9]. The study describes the clinical manifestations, radiological examination and gene mutation analysis findings and rhGH treatment responses in patients with syndromic short stature.

## Methods

### Patients

Patients were referred to the paediatric department of Beijing Jishuitan Hospital from March 2017 to December 2019. The criteria for the diagnosis of syndromic short stature were as follows [10–12]: height two standard deviations (SDs) below the mean of healthy Chinese children adjusted for age and sex; the presence of multiple malformations; and/or intellectual disability. The exclusion criteria were as follows: chronic liver and renal disease and comorbidities that could affect growth. These patients had unrecognized syndromes based on clinical and radiological manifestations. Written informed consent was obtained from the guardians of these patients. Clinical data and peripheral lymphocyte DNA were collected from the probands and their parents. All the probands had normal G-banded karyotyping.

### Trio-WES

Trio-WES was used to identify pathogenic gene mutations in patients with short stature. Genomic DNA was extracted from peripheral blood using the Blood Genome Column Medium Extraction Kit (Kangweishiji, China) according to the manufacturer’s instructions. The extracted DNA samples were subjected to quality control using a Qubit 2.0 fluorimeter and electrophoresis with a 0.8% agarose gel for further protocols. Protein-coding exome enrichment was performed using xGen Exome Research Panel v1.0 (IDT, Iowa, USA), which consists of 429,826 individually synthesized and quality-controlled probes, targeting a 39 Mb protein-coding region (19,396 genes) of the human genome and covering 51 Mb of end-to-end tiled probe space. High-throughput sequencing was performed by an Illumina NovaSeq 6000 series sequencer (PE150), and no less than 99% of the target sequences were sequenced. The sequencing process was performed by Beijing Chigene Translational Medicine Research Center. Raw data were processed quickly for adapter removal and low-quality read filtering. The paired-end reads were performed using Burrows–Wheeler Aligner to the Ensemble GRCh37/hg19 reference genome. Base quality score recalibration, together with single nucleotide polymorphisms (SNPs) and short indel calling, was conducted using GATK. According to the sequence depth and variant quality, SNPs and indels were screened, and high-quality and reliable variants were obtained. The online system independently developed by Chigene (www.chigene.org) was used to annotate database-based minor allele frequencies (MAFs) and American College of Medical Genetics (ACMG) practice guideline-based pathogenicity of every yielded gene variant, and the system also provides a serial software package for conservative analysis and protein product structure prediction.

The databases for MAF annotation include the 1000 Genomes, dbSNP, ESP, ExAC, and Chigene in-house MAF databases. The Provean, Sift, Polypen2_hdiv, Polypen2_hvar, MutationTaster, M-Cap, and Revel software packages were used to predict protein product structure variation. As a prioritized pathogenicity annotation to ACMG guidelines, Online Mendelian Inheritance in Man (OMIM), Human Gene Mutation Database (HGMD) and ClinVar databases were used as conferences of pathogenicity of every variant [13]. Sanger sequencing was further used to confirm the candidate variants. The patients’ parents were also examined to determine the origins.

### Whole-genome low-coverage sequencing

Whole-genome low-coverage sequencing was performed to identify CNVs in patients with syndromic short stature and intellectual disability. Genomic DNA was extracted from peripheral blood samples and sheared to 200–300 bp fragments by sonication, followed by electrophoresis analysis for quality control. The fragments were then end-repaired and A-tailed in preparation for ligation to adapters. The ligates were amplified by ligation-mediated PCR for 4–6 rounds. High-throughput sequencing was performed on an Illumina NovaSeq 6000 series sequencer (Illumina, San Diego, CA, USA). Raw image files were processed by BclToFastq (Illumina) for base calling and raw data generation. Reads were mapped to reference genome hg19 using BWA software. CNVs of 100 kb or more in length were detected using Chigene independently developed software packages for CNV detection.

CNV intervals were detected and screened according to public CNV databases and local databases. Decipher, ClinVar, ClinGen, and the Database of Genomic Variants (DGV) were used as references to annotate the pathogenic classification of each screened CNV. The biological harm and related phenotypes of CNVs were assessed by annotated information and frequency databases according to the ACMG practice guidelines and 2011 and 2013 CNV diagnostic guidelines [14, 15].

### GH treatment

GH stimulation tests were performed to confirm whether patients had GH deficiency. Oral clonidine (4 µg/kg) or 10% arginine (0.5 g/kg, maximum dose 30 g) intravenous injection was administered. Then, blood samples were drawn at 0′, 30′, 60′, 90′, and 120′ time points to examine GH concentration. Normal GH was defined as a GH level above 10 ng/mL, partial GH deficiency was defined as a GH level of 5–10 ng/mL, and complete GH deficiency was defined as a GH level below 5 ng/mL. According to the growth pattern and bone age (BA) delay, rhGH (GenSci, China) treatment was initiated to improve height. Patient height and weight were measured and recorded every 3 months. The patient’s biochemical parameters, such as liver and kidney functions, myocardial enzymes, blood electrolytes, blood glucose, haemoglobin A1c (HbA1C), insulin (INS) and thyroid function, were also monitored every 3 months. Their BA was examined every six months to one year. The adverse effects of GH therapy, such as headaches, hypertension, arthralgia, liver damage, tumours, hypothyroidism, and diabetes, were carefully noted. GH dosage was adjusted according to the growth velocity and insulin-like growth factor-1 (IGF-1) level.

## Results

All methods were carried out in accordance with relevant guidelines and regulations. Seven patients with syndromic short stature were recruited in this study, as shown in Table 1. In addition to short stature, other features were skeletal dysplasia (n = 7), facial dysmorphism (n = 5), intellectual disability (n = 3), and congenital heart disease (n = 1). We identified 2 pathogenic mutations (*FGFR3*, c.1620C>A p.N540K; *TRPS1*, c.2527_c.2528dupTA p.S843fsX72) and 1 likely pathogenic mutation (*GNAS*, c.2288C>T p.A763V) in three patients, respectively. All of these mutations were heterozygous and inherited from their affected parents. This *FGFR3* mutation has been reported previously, and the two other mutations are novel.

**Table 1:** Clinical phenotypes and mutation analysis in seven patients with syndromic short stature

Patient	Year	Sex	Technique	Short stature	Intellectual disability	Facial dysmorphism	Other features	Result	Diagnosis	GH therapy
1.	4–3/12	Female	Trio-WES, CNVs	+	+	Strabism	Macrocephaly, short limbs, depigmentation, PHPV of right eye	*FGFR3,* NM_000142.4, c.1620C>A (p.N540K) *ZNF687*, NM_020832.2, c.2810C>G (p.P937R)	Hypochondroplasia	Yes
2.	6	Female	Trio-WES, CNVs	+	+	−	Bilateral asymmetry, café au lait macule, congenital heart disease	*COL1A2*, NM_000089, c.2943+21C>T	VUS	No
3.	5	Male	Trio-WES	+	−	Triangle face, facial asymmetry	Bilateral asymmetry, hypertonia of left limbs, muscle weakness and atrophy of left limbs, inguinal hernia, obstructive sleep apnoea	*TRPV4*, NM_021625, c.2572C>T (p.Q858X)	VUS	No
4.	12–9/12	Female	Trio-WES, CNVs	+	+	Triangle face	Brachydactyly, brachymetacarpia	*GNAS*, NM_080425, c.2288C>T (p.A763V)	PHP-Ia	Yes
5.	10–7/12	Male	Trio-WES	+	−	Macrocephaly, hypertelorism, epicanthal folds, anteverted nares, mid-face hypoplasia, thick lips, supernumerary teeth	Open fontanelles, drooping shoulders, hypoplastic clavicles, narrow thorax	*COL11A1*, NM_001854.3, c.C5095G (p.L1699V); *GLI3*, NM_000168.5, c.A4721G (p.K1574R)	VUS	No
6.	1–3/12	Female	Trio-WES	+	−	Sparse hair, bulbous tip of the nose	Cone-shaped epiphyses, severe eczema	*TRPS1*, NM_014112, c.2527_c.2528dupTA (p.S843fsX72)	TRPS I	No
7.	2–11/12	Male	Trio-WES	+	−	−	3–5th Distal phalanges hypoplasia of left hand, simian crease, multiple dental caries, depigmentation, eczema	−	Unknown	No

**Fig. 1:**
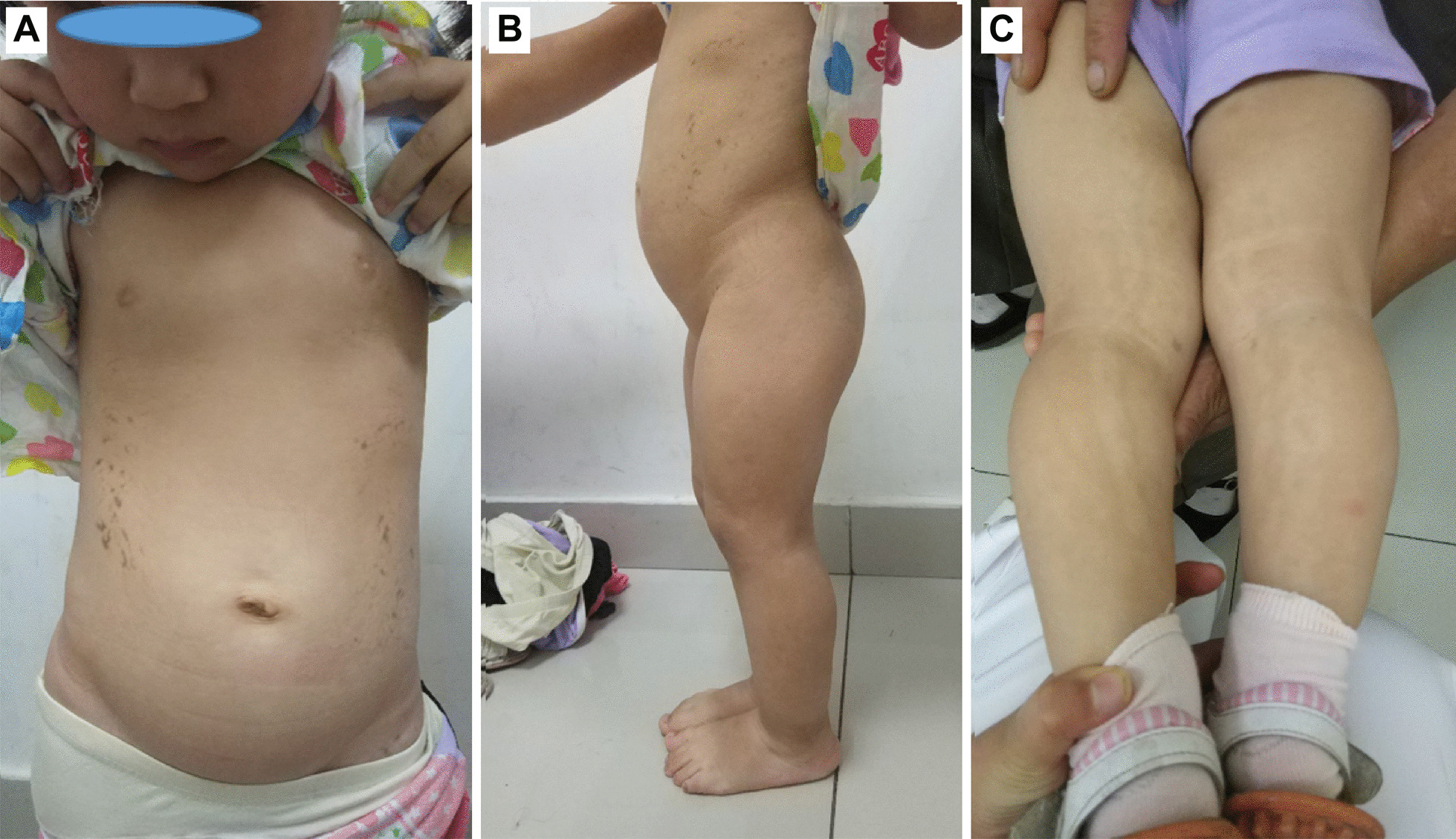
Clinical features of the proband. **A** Brown spots on both sides of abdomen. **B** Lumbar lordosis with protruding abdomen. **C** Tibial bowing and linear depigmentation in both lower limbs

**Fig. 2:**
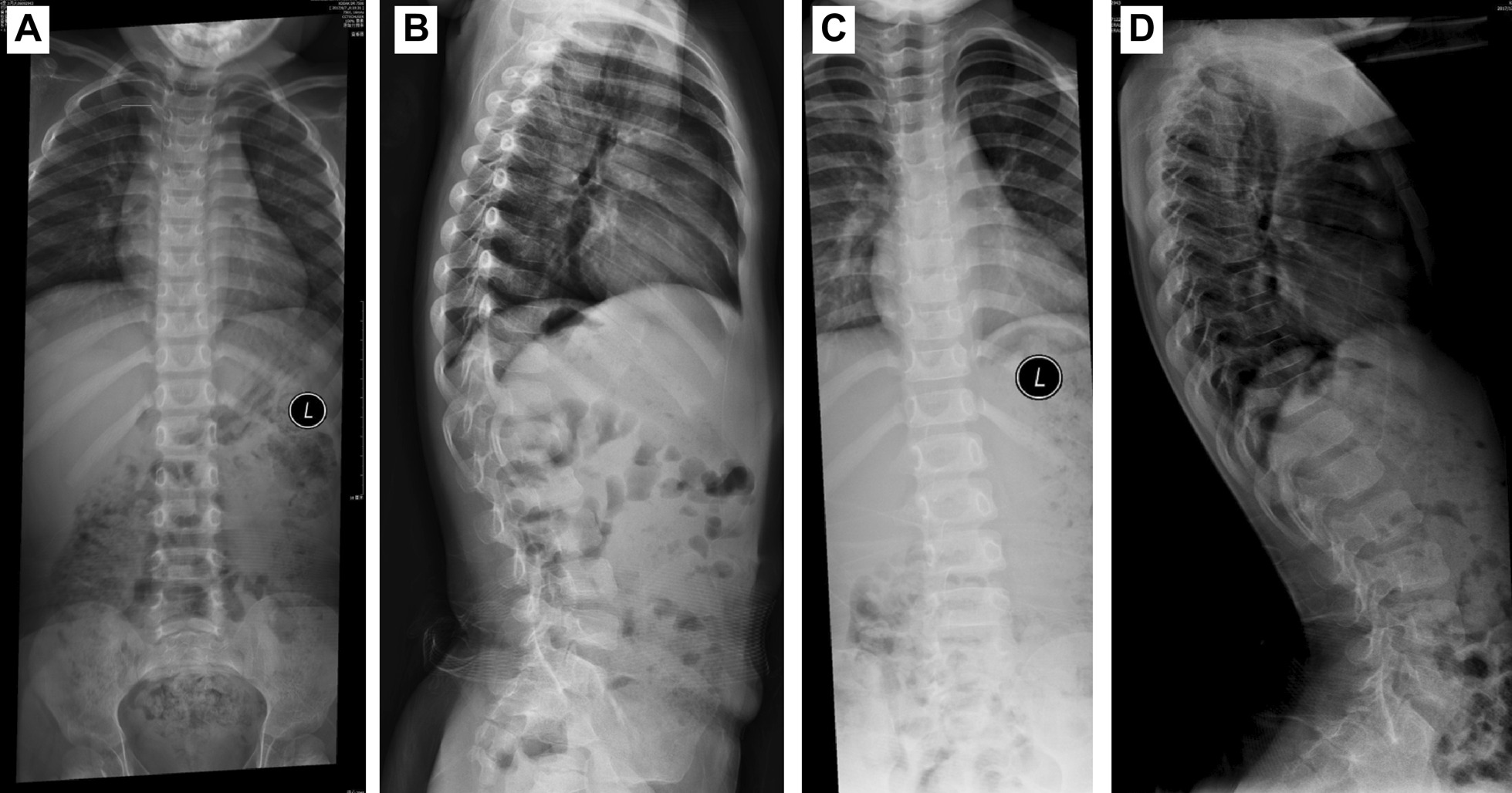
Frontal and lateral spinal imaging data of the proband with hypochondroplasia. She had thoracic kyphosis and dorsal concavity of the lumbar vertebrate bodies. The interpedicular distance widened in the lower lumber spine more than in the upper lumber spine when she was 4–3/12 years (**A**, **B**) and 4–9/12 years (**C**, **D**)

**Fig. 3:**
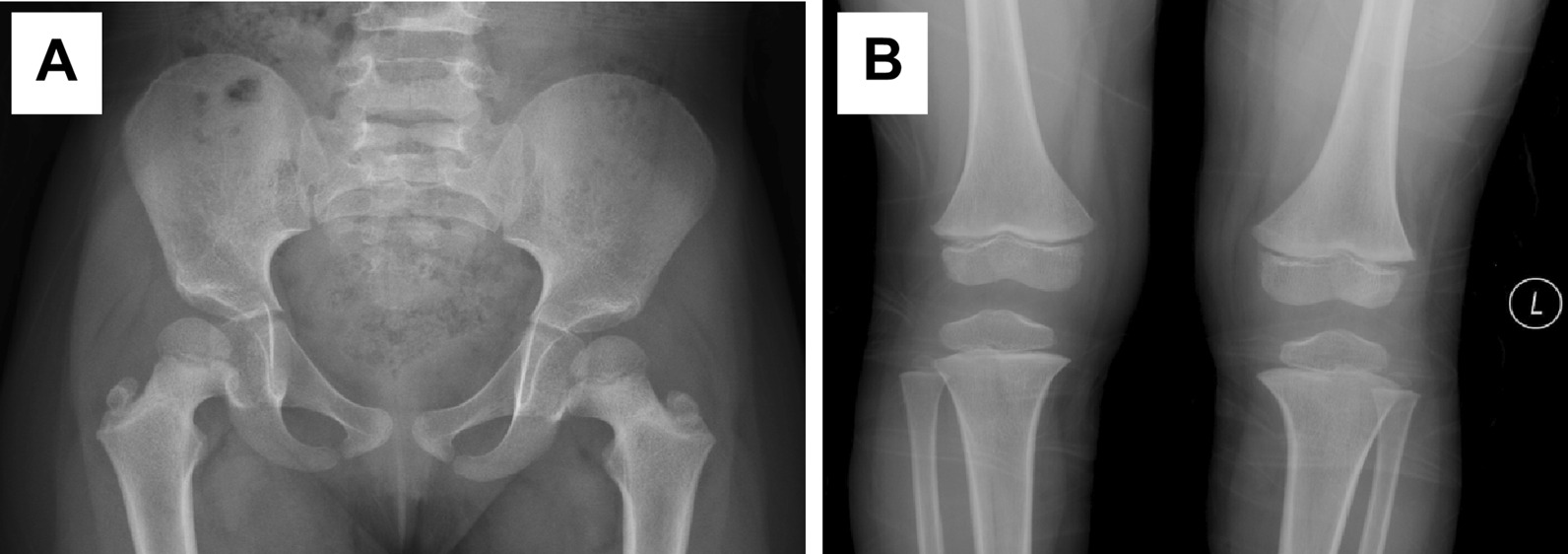
**A** Short, broad femoral neck; short, squared ilea; and flattened acetabular roof (5 years). **B** Shortening of the long bones with metaphyseal flare (5–7/12 years)

The first proband was a 4- and 3/12-year-old girl. She had short stature and short limbs. She had intellectual disability, persistent hyperplastic primary vitreous (PHPV) of the right eye, and incontinentia pigmenti. Other clinical manifestations included macrocephaly, lumbar lordosis with protruding abdomen, genu varum, tibial bowing, and joint laxity (Fig. 1). She was born at full term by caesarean section. Her birth weight was 2750 g, and her birth length was unknown. She had no history of perinatal asphyxia. Her height was 85.8 cm (− 5.05 SD), weight was 13.8 kg (− 1.74 SD), and body mass index (BMI) was 18.75 kg/m^2^ (2.38 SD). Her head circumference was 53 cm (2.61 SD). Her upper segment was 51.5 cm, and her lower segment was 34.5 cm. The upper/lower segment ratio was 1.5 (normal: 1.23–1.47). Her radiological features included dorsal concavity of the lumbar vertebrate bodies, widened inferior lumber interpedicular distance, a short and broad femoral neck, short squared ilea, a flattened acetabular roof, and a shortening of the long bones with metaphyseal flare, as shown in Figs. 2 and 3 [16, 17].

Both her father and mother had intellectual disability, with heights of 150 cm and 151 cm, respectively. A fibroblast growth factor receptor 3 (*FGFR3*) mutation c.1620C>A (p.N540K) was identified in the proband and her father [18].

**Fig. 4:**
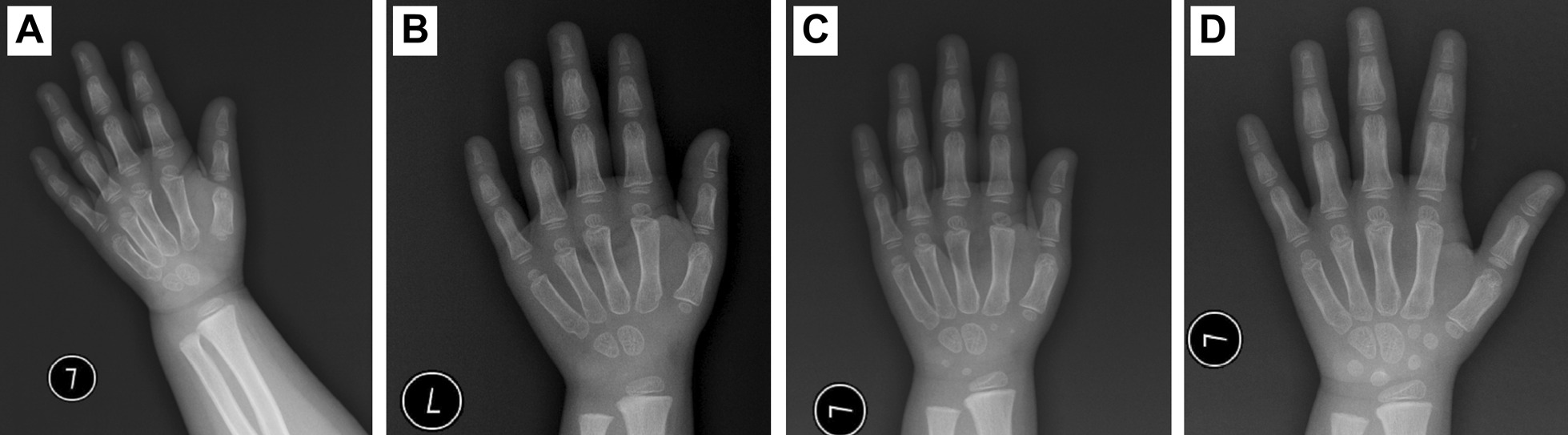
Bone age radiograph in the course of GH treatment. **A** CA: 4–3/12 years; BA: 2.5 years. **B** CA: 4–9/12 years; BA: 3 years. **C** CA: 5–4/12 years; BA: 4 years. **D** CA: 6–4/12 years; BA: 5 years

**Fig. 5:**
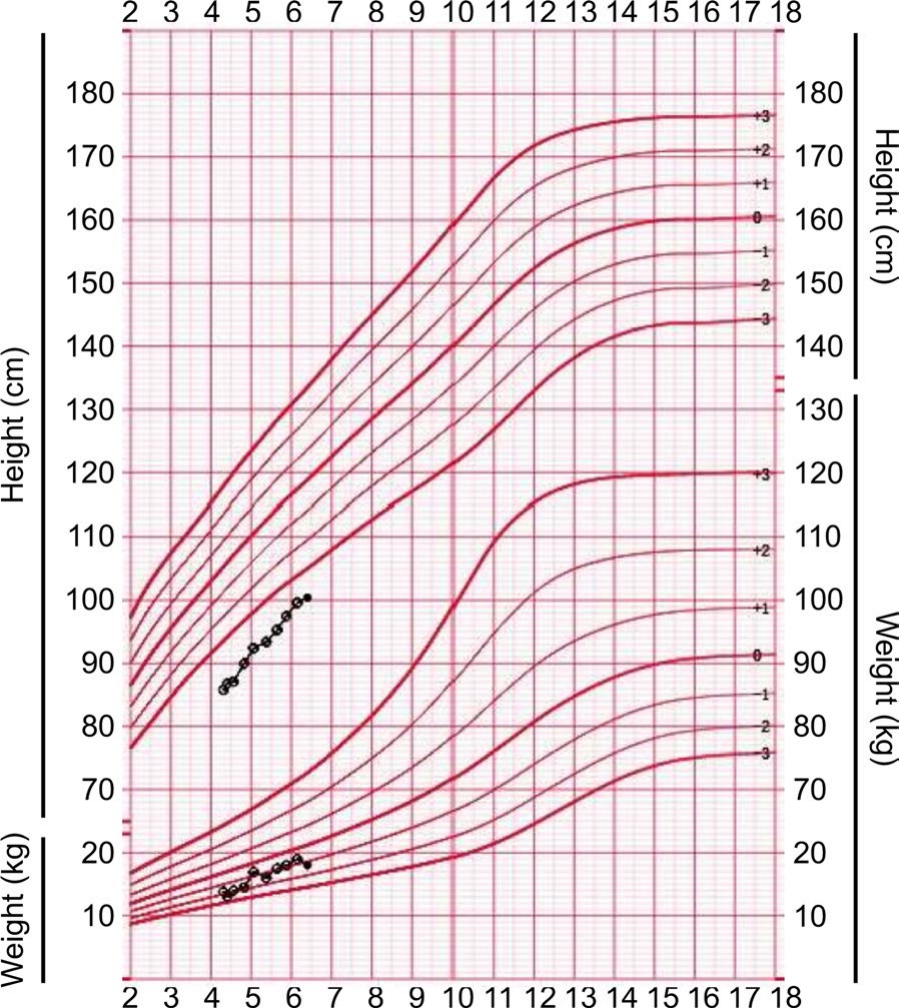
Growth curves of height and weight in the first proband after GH therapy

GH provocative tests showed peak concentrations of 8.42 ng/mL after levodopa and 8.21 ng/mL after arginine. At presentation, the patient’s height was 85.8 cm, − 5.05 SD height for age. Although her chronological age (CA) was 4–3/12 years, her BA was 2.5 years. Before treatment, the mean annual growth velocity of the patient was 3–4 cm. Long-acting GH (polyethylene glycol recombinant human somatropin injection) was initiated as shown in Table 2. After 25 months of treatment, her height improved to 100.4 cm, − 4.02 SD height for age, and her increased height SD score (SDS) was 1.03. Her BA advanced from 2.5 to 5 years, as shown in Fig. 4. Her growth velocity increased by 7.6 cm in the first year and 7.0 cm in the second year. In the first year of treatment, her height improved to 93.4 cm (− 4.59 SD height for age), and her increased height SDS was 0.46, as shown in Fig. 5. Because of the low IGF-1 level and decreased growth velocity, the dosage was increased from 0.2 mg/kg/w when she was 5 and 7/12 years to 0.3 mg/kg/w when she was 6 and 1/12 years. In the second year, her height reached 100.4 cm, − 4.02 SD height for age, and her increased height SDS was 0.57. Her biochemical parameters, blood glucose, and HbA1c were normal in the follow-up period. She had no manifestations of GH adverse effects. However, she had high IGF-1 and FT4 levels at a dose of 0.3 mg/kg/week. At 6 and 4/12 years, she stopped GH treatment because of economic factors.

**Table 2:** Clinical parameters of the first proband after GH treatment

CA (year)	BA (year)	Height (cm)	Weight (kg)	GH (mg/kg/w)	IGF-1 ng/mL	HbA1c (3.6–6.0%)	INS (2.6–11.8 µU/mL)	T3 (nmol/L)	T4 (nmol/L)	FT3 (pmol/L)	FT4 (pmol/L)	TSH (mIU/L)
4–3/12	2.5	85.8 (− 5.05SD)	13.8 (− 1.74SD)	0.2	88.9 (49–283)	4.7	1.8	3.0 (1.4–3.8)	120.3 (76.6–189.0)	7.1 (3.7–8.5)	17.8 (12.3–22.8)	2.91 (0.7–5.97)
4–4/12		86.8 (− 4.93SD)	13.0 (− 2.38SD)	0.2	124 (49–283)	5.2	/	3.4 (1.4–3.8)	145.9 (76.6–189.0)	7.9 (3.7–8.5)	21.0 (12.3–22.8)	2.95 (0.7–5.97)
4–6/12		87.1 (− 5.07SD)	14.0 (− 1.86SD)	0.2	93.9 (49–283)	4.8	4.0	3.1 (1.4–3.8)	112.1 (76.6–189.0)	8.1 (3.7–8.5)	18.3 (12.3–22.8)	2.72 (0.7–5.97)
4–9/12	3	90.0 (− 4.71SD)	40.5 (− 1.81SD)	0.2	107 (49–283)	5.2	2.7	2.9 (1.4–3.8)	142.2 (76.6–189.0)	6.7 (3.7–8.5)	20.1 (12.3–22.8)	2.30 (0.7–5.97)
5		92.4 (− 4.42SD)	16.95 (− 0.67SD)	0.2	245 (50–286)	4.5	2.9	3.4 (1.4–3.8)	123.8 (76.6–189.0)	8.2 (3.7–8.5)	19.2 (12.3–22.8)	4.19 (0.7–5.97)
5–4/12	4	93.4 (− 4.59SD)	16.0 (− 1.44SD)	0.2	291↑ (50–286)	5.3	4.9	3.2↑ (1.3–3.1)	154.9 (66.0–181.0)	8.1 (3.0–9.1)	22.4↑ (12.0–22.0)	1.93 (0.27–4.2)
5–7/12		95.3 (− 4.46SD)	17.5 (− 0.93SD)	0.3	149 (50–286)	5.1	2.1	3.3↑ (1.3–3.1)	157.0 (66.0–181.0)	8.3 (3.0–9.1)	23.1↑ (12.0–22.0)	3.21 (0.27–4.2)
5–10/12	4.5	97.5 (− 4.19SD)	18.0 (− 0.88SD)	0.3	473↑ (50–286)	5.3	9.1	3.6 (1.3–3.1)	171.4 (66.0–181.0)	8.3 (3.0–9.1)	27.2↑ (12.0–22.0)	1.46 (0.27–4.2)
6–1/12		99.6 (− 3.96SD)	19.0 (− 0.65SD)	0.2	416↑ (52–297)	5.1	5.6	3.7 (1.3–3.1)	148.0 (66.0–181.0)	8.6 (3.0–9.1)	22.1↑ (12.0–22.0)	1.61 (0.27–4.2)
6–4/12	5	100.4 (− 4.02SD)	18.0 (− 1.25SD)	0.2	304 (52–297)	5.1	4.2	3.2↑ (1.3–3.1)	128.7 (66.0–181.0)	7.7 (3.0–9.1)	19.4 (12.0–22.0)	1.87 (0.27–4.2)

T3 triiodothyronine, T4 thyroxine, FT3 free triiodothyronine, FT4 free thyroxine, TSH thyroid-stimulating hormone

**Fig. 6:**
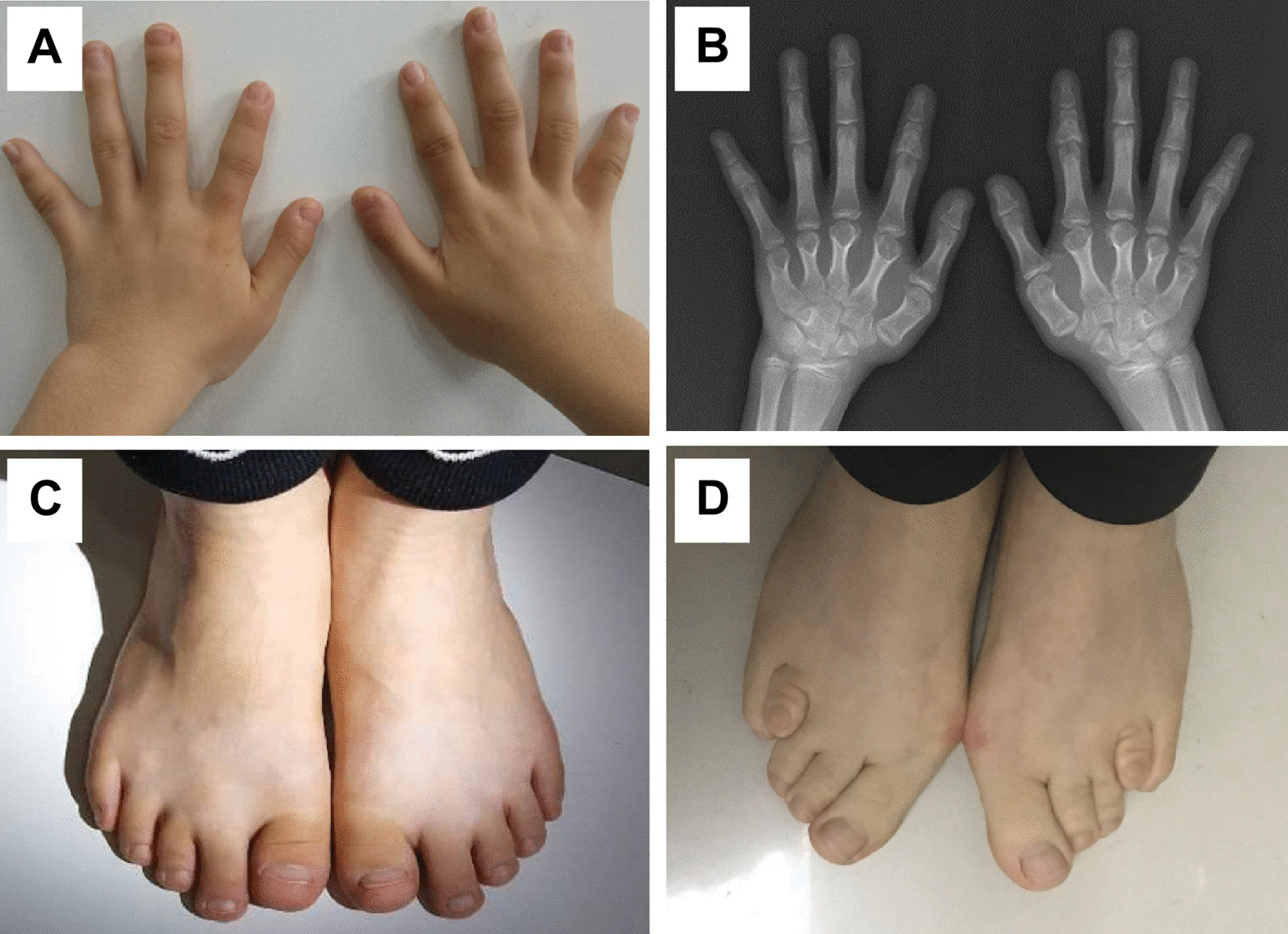
Clinical features and radiological manifestations of the fourth pedigree. **A**, **B** The proband had a shortening of the metacarpals, distal phalanx of the thumb and middle figure, middle phalanx of the index figure and the fifth finger. **C** The proband had short feet and brachydactyly. **D** Her mother had a shortening of the 4th metatarsals

The fourth proband was a 12- and 9/12-year-old girl. She had short stature with brachydactyly, as shown in Fig. 6. Her growth velocity was 2–3 cm per year. Her height was 139.3 cm (− 2.65 SD), her weight was 30.4 kg (− 2.26 SD), and her BMI was 15.67 kg/m^2^ (− 1.12 SD). Her pubertal stage was breast Tanner II and pubic hair Tanner I. She was born at full term by caesarean section. She had no history of perinatal asphyxia or difficulty feeding after birth. Her milestones were normal. She had mild intellectual disability and poor academic performance. She exhibited normal liver and kidney functions, myocardial enzymes and blood electrolytes. Indicators of bone metabolism were as follows: Ca 2.33 (2.25–2.67) mmol/L, P 1.59 (1.45–2.10) mmol/L, ALP 256 (50–400) IU/L, and 25-hydroxy-vitamin D_3_ (25-(OH)VD_3_) 22.42 (30–100) ng/mL. She had normal thyroid function at the first visit, as shown in Table 3. The GH concentration response to an exercise test was 23.10 ng/mL. Her IGF-1 was 259 (143–693) ng/mL. Her BA was equivalent to approximately 13.5 years (Fig. 6B) at a CA of 12 and 9/12 years. Her pituitary magnetic resonance imaging (MRI) findings were normal. Her parents were nonconsanguineous. Her mother’s height was 146.7 cm, her weight was 51.5 kg, and her BMI was 23.9 kg/m^2^. Her mother had short stature, a round face, obesity, and a shortening of the 4th metatarsals (Fig. 6D). The mother refused to undergo a blood test, so her endocrine hormone levels were unknown. The father and older brother of the proband had normal phenotypes; their heights were 170 cm and 165 cm, respectively.

**Table 3:** BA, height, weight, IGF-1, HbA1c and thyroid function in the fourth proband after GH treatment

CA (year)	BA (year)	Height (cm)	Weight (kg)	GH (IU/kg/d)	IGF-1 ng/mL	HbA1c (3.6–6.0%)	INS (2.6–11.8 µU/mL)	FT3 (pmol/L)	FT4 (pmol/L)	TSH (mIU/L)	Levothyroxine (µg)
12–9/12	13.5	139.3 (− 2.69SD)	30.4 (− 2.30SD)	0.15	259 (143–693)	5.2	6.9	6.3 (3.5–7.7)	15.3 (12.0–22.0)	4.19 (0.27–4.20)	
13–1/12		140.2 (− 2.73SD)	33.4 (− 1.88SD)	0.15	361 (183–850)	5.2	10.0	6.4 (3.5–7.7)	12.3 (12.0–22.0)	6.39↑ (0.27–4.20)	
13–7/12	14.5	144.0 (− 2.38SD)	34.3 (− 2.04SD)	0.16	503 (183–850)	5.2	6.6	8.0 (3.5–7.7)	15.4 (12.0–22.0)	7.07↑ (0.27–4.20)	25
13–11/12		145.0 (− 2.36SD)	38.5 (− 1.43SD)								

**Fig. 7:**
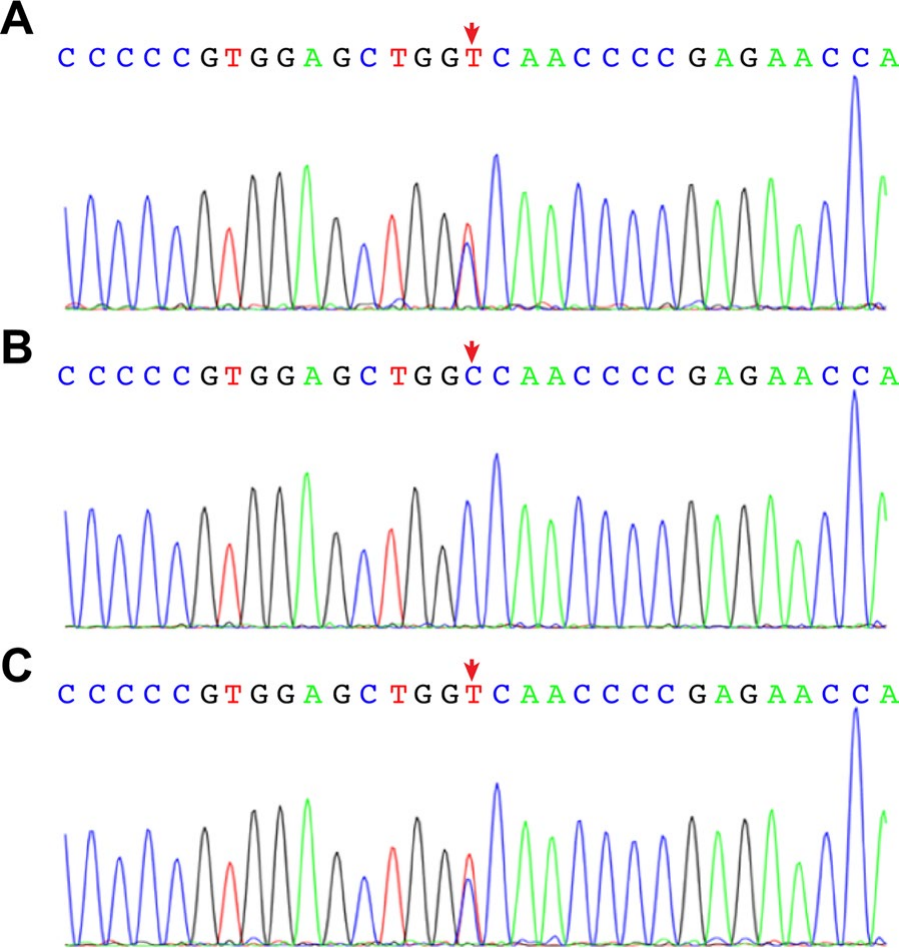
Sanger sequencing of *GNAS* in the fourth pedigree 4: **A** the proband, **B** her father, and **C** her mother. The proband and her mother had the *GNAS* mutation c.2288C>T p.A763V. The mutation is indicated by arrows

Trio-WES identified the *GNAS* variant c.2288C>T p.A763V in the proband and her mother (Fig. 7). Bioinformatics analysis suggested that this variant was located in a highly conserved domain and was likely a pathogenic mutation. This mutation could cause *GNAS* inactivation. The paternal origin of the *GNAS* mutation in the mother was uncertain because of specimens from the proband’s grandfather and grandmother were not available.

**Fig. 8:**
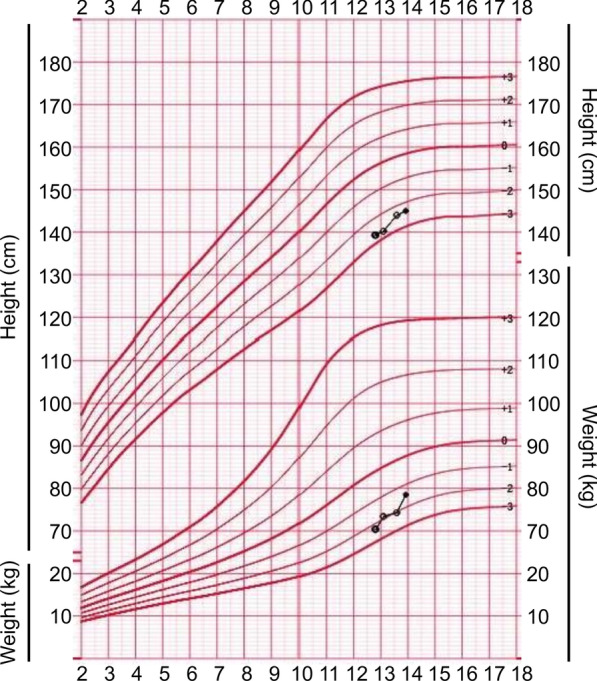
Growth curves of height and weight in the fourth proband after GH therapy

Substitutive short-acting rhGH treatment was started, as shown in Table 3. After 13 months, her height improved from 139.3 cm (− 2.69 SD) to 145.0 cm (− 2.36 SD), as shown in Fig. 8. Her growth velocity increased from 2–3 to 4.75 cm/year. During treatment, she had elevated TSH levels, and subclinical hypothyroidism was diagnosed. Then, thyroxine 25 μg was administered daily. Her biochemical parameters, blood glucose, and HbA1c remained normal. She had no other adverse reactions. When she was 13 and 7/12 years, her BA was 14.5 years. Her intact parathyroid hormone (iPTH) level was 66.6 (15–65) pg/mL, while her 25-(OH)VD_3_ level was 32.88 (30–100) ng/mL. Indicators of bone metabolism were normal: Ca 2.38 (2.25–2.67) mmol/L, P 1.85 (1.45–2.10) mmol/L, and ALP 298 (50–400) IU/L. Her sex hormones were as follows: follicle-stimulating hormone (FSH) 9.00 IU/L, luteinizing hormone (LH) 2.03 IU/L, prolactin (PRL) 126.26 mIU/L, progesterone (P) 0.72 nmol/L, oestradiol (E2) 277.0 pmol/L, and testosterone (T) 2.36 nmol/L. The patient stopped rhGH at the age of 13 and 11/12 years. She had menarche at 14–3/12 years.

**Fig. 9:**
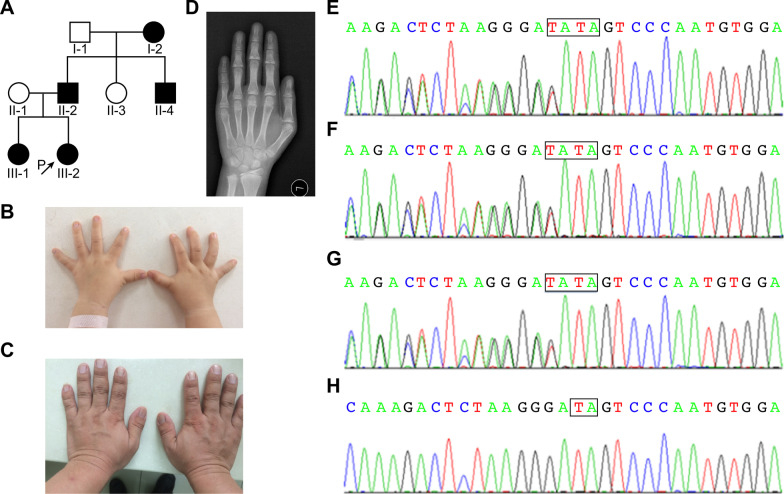
Clinical manifestations of the sixth pedigree. **A** Pedigree tree of the family with TRPS I. The proband (**B)** and her father (**C**) had short and stubby hands. **D** Bone age radiograph of the patient’s older sister showed the cone-shaped epiphyses with a shortening of the middle phalanxes of the second to fifth fingers. The *TRPS1* mutation c.2526_c.2527dupTA (p. S843fsX72) was detected in the three affected patients: **E** the proband, **F** her older sister, **G** her father, and **H** her mother

The sixth proband was a 15-month-old girl. Her clinical manifestations included short stature, sparse and fine hair, a bulbous nose tip, a long philtrum, a thin upper vermilion, a prominent forehead and cone-shaped phalangeal epiphyses (Fig. 9). Her height was 72.6 cm (− 2.27 SD), weight was 8.78 kg (− 1.33 SD), and BMI was 16.66 kg/m^2^ (0.31 SD). The patient was born at full term by caesarean section. Her birth weight was 2480 g (− 2.19 SD), and her birth length was 46 (− 2.22 SD) cm. She was followed up in our clinical department. When she was 2 and 1/12 years, her height was 81.6 cm (− 1.94 SD), weight was 10.3 kg (− 1.56 SD), BMI was 15.7 kg/m^2^ (− 0.14 SD), and head circumference was 48 cm (0.41 SD). Her IGF-1 was 73.5 (51–303) ng/mL. She had normal thyroid function.

There were five affected patients in three generations in an autosomal-dominant manner, as shown in Fig. 9. The proband’s father and older sister had similar manifestations: sparse hair, a bulbous nose tip, a long philtrum, a thin upper vermilion, and cone-shaped phalangeal epiphyses. In addition to these specific features, her father also had overcrowded teeth. The height of the affected father (II-1) and uncle (II-4) was approximately 160 cm. The proband’s grandmother (I-2) was approximately 150 cm in height. The proband’s older sister was 10 and 7/12 years old at presentation. She had been diagnosed with central precocious puberty (CPP) and received leuprorelin (gonadotropin releasing hormone agonist, GNRHa) therapy for 28 months since the age of 8 and 3/12 years in another hospital. Her height was 145.6 cm (0.19 SD), and her weight was 34.3 kg (− 0.03 SD). She was born at full term by caesarean section. Her birth weight was 2750 g (− 1.30 SD), and her birth length was 48 cm (− 1.01 SD). When she was 10 and 11/12 years old and her BA was almost 11 years (Fig. 9D), leuprorelin was withdrawn. At 11 and 3/12 years old, her sex hormones were normal: FSH 6.64 IU/L, LH 3.80 IU/L, PRL 226.62 mIU/L, P 0.51 nmol/L, E2 186 pmol/L, and T 1.70 nmol/L. The serum IGF-1 was 390 (111–551) ng/mL. When she was 14 and 4/12 years old, her near-adult height reached 160 cm (0.19 SD). Her mother’s height was 140 cm, which was caused by scoliosis after a traffic accident. A *TRPS1* mutation (c.2526_c.2527dupTA (p. S843fsX72) in exon 5 was detected in the affected patients of the pedigree (Fig. 9).

The third proband had short stature, bilateral asymmetry, muscle atrophy of the left limbs, and an inguinal hernia. One novel *TRPV4* mutation (c.2572C>T p.Q858X) was identified in the patient and her unaffected father; however, the phenotype–genotype correlation was uncertain [19–21]. One novel *COL11A1* variation (c.C5095G p. L1699V) and one novel *GLI3* variation (c.A4721G p.K1574R) was identified in the fifth proband with cranial dysplasia, clavicle hypoplasia and supernumerary teeth, which were the main features of cleidocranial dysplasia [22, 23]. Both were variants of uncertain significance (VUSs) (http://wintervar.wglab.org/ and http://varcards.biols.ac.cn/).

## Discussion

We performed Trio-WES analysis in seven patients with syndromic short stature of unknown aetiology and Trio-WES and CNV combined analysis in three patients with concurrent intellectual disability. One *FGFR3* mutation (c.1620C>A p.N540K), one likely pathogenic *GNAS* mutation (c.2288C>T p.A763V), and one *TRPS1* mutation (c.2527_c.2528dupTA p.S843fsX72) was identified in three pedigrees with hypochondroplasia, pseudohypoparathyroidism Ia (PHP-Ia), and trichorhinophalangeal syndrome type I (TRPS I). Our findings emphasize the importance of Trio-WES analysis in identifying the aetiology of syndromic short stature.

### Hypochondroplasia

*FGFR3*, located at 4p16.3, is a receptor tyrosine kinase and a member of the fibroblast growth factor receptor family with a highly conserved structure. *FGFR* genes are characterized by an extracellular ligand-binding domain consisting of three immunoglobulin (Ig) subdomains, a transmembrane domain, and a split intracellular tyrosine kinase domain [24]. *FGFR3* mutations have been reported to cause various types of skeletal dysplasia, especially achondroplasia and hypochondroplasia, in an autosomal-dominant manner [25]. The most common and specific *FGFR3* mutation in achondroplasia is c.1138G>A or c.1620G>C (p.G380R) and in hypochondroplasia is c.1620C>A or c.1620C>G (p.N540K) [16, 26]. Hypochondroplasia has many features that are similar to achondroplasia, but hypochondroplasia is milder. The effects of *FGFR3* mutation have been shown to result in constitutive activation of receptor tyrosine kinase and negative regulation of cartilage growth [16].

Hypochondroplasia is a genetic skeletal dysplasia characterized by disproportionate short stature, lumbar lordosis, stocky build, short arms and legs, and macrocephaly [16, 27]. The diagnosis of hypochondroplasia is based on clinical manifestations, radiological features and *FGFR3* mutation analysis. The proband had short stature, macrocephaly, body disproportion, lumbar lordosis, and joint laxity. However, the coexistence of intellectual disability and PHPV made the diagnosis complicated. An *FGFR3* mutation c.1620C>A (p.N540K) was identified in the proband and her father. Both of them had intellectual disability, which is more frequent in hypochondroplasia than in achondroplasia [16, 28]. In addition, unlike achondroplasia, the proband and her affected father had normal faces and lacked trident hands. They were diagnosed as having hypochondroplasia. The PHPV in the proband may be caused by other unidentified gene mutations.

As the proband had concurrent partial GH deficiency, she had severe short stature. Similar to other studies, the proband had a good response to GH therapy [27]. GH improved her height from 85.8 cm (− 5.05 SD) to 100.4 cm (− 4.02 SD), and her increased height SDS was 1.03 after 25 months of treatment. Her growth velocity and IGF-1 level decreased in the second year; however, an increase in GH could maintain her growth velocity. GH therapy should be considered to increase the final adult height in patients with this growth disorders [18, 29]. Data about final height in the proband should be followed up.

### PHP

*GNAS* encodes the alpha subunit of the stimulatory guanine nucleotide-binding protein, which is downstream of many different G protein-coupled hormone receptors [30]. Disorders of *GNAS* inactivation include various phenotypes, including PHP Ia, Ib, and Ic; pseudoPHP (PPHP); progressive osseous heteroplasia (POH), and osteoma cutis (OC) [31]. The specific phenotype was determined by the mutation type and parental origin of the affected allele [32]. PHP-Ia is caused by maternal heterozygous *GNAS* mutations and characterized by multiple hormonal resistance and characteristic features of Albright’s hereditary osteodystrophy (AHO) [32].

The proband and her mother had short stature and brachydactyly. Both the proband and her mother were positive for the *GNAS* likely pathogenic mutation c.2288C>T p.A763V. *GNAS*, located at the *GNAS* complex locus at 20q13.32, is encoded by exons 1–13. *GNAS* mutation c.2288C>T p.A763V, located in exon 5, could affect the structure and function of GNAS and neuroendocrine secretory protein 55 (NESP55) in tissues with maternal-specific expression, such as the renal proximal tubules, thyroid, gonads, and pituitary [30]. This heterozygous mutation can also cause haploinsufficiency in biallelically expressed tissues such as bone and growth plate chondrocytes [30].

The diagnosis of PHP was based on clinical manifestations, endocrine hormone levels and *GNAS* gene testing. The proband had short stature, brachydactyly, multiple hormone resistance, and mild intellectual disability. She was characterized by PTH resistance and TSH resistance. PTH was inappropriately elevated considering normal calcium and phosphate levels, which indicates the peripheral resistance of PTH. She had TSH resistance with high TSH levels and normal thyroid hormone levels. Her mother’s manifestations were consistent with AHO: short stature, a round face, and brachydactyly type E (shortening mainly of the 4th metatarsals). The proband and her mother show obvious phenotypic heterogeneity.

Because of the low normal IGF-1 level, especially the lack of a pubertal growth spurt, linear growth attenuation in the proband worsened after 10 years. Her height SDS increased from − 2.69 to − 2.36 after 13 months of GH therapy. During GH treatment, she had a high TSH level, and levothyroxine was administered. There were no other side effects, indicating that GH was effective and safe for this PHP-Ia patient. The observations in this proband were consistent with a previous study showing that GH therapy had positive effects on prepubertal children with PHP-Ia [33]. The height changes, benefits, and side effects during GH treatment need to be monitored in a large sample of individuals with this rare disease.

### TRPS I

TRPS I is characterized by craniofacial and skeletal malformations, including short stature, sparse hair, a bulbous nose, a long flat philtrum, a thin upper vermilion border, protruding ears, cone-shaped phalanges and hip dysplasia [34]. The disorder is caused by *TRPS1* haploinsufficiency. *TRPS1* is located at 8q23.3 and encodes a zinc-finger transcription repressor involved in the regulation of chondrocyte and perichondrium development [35]. We report here a family with a novel *TRPS1* mutation c.2526_c.2527dupTA (p.S843fsX72). The father and his two daughters carried this mutation and had clinical manifestations of TRPS I.

The insertion frameshift mutation c.2526_c.2527dupTA (p. S843fsX72) in *TRPS1*, which leads to the absence of the GATA DNA-binding zinc finger, potential nuclear localization signals and IKAROS-like zinc-finger motif, was predicted to result in a loss of function in transcription [36, 37]. The affected patient in our study had mild short stature and brachydactyly, consistent with other studies showing that most patients with *TRPS1* nonsense mutations have a less severe phenotype [37, 38].

The GH response in patients with TRPS I has been inconsistent according to previous studies [34]. The proband and her older sister had clinical manifestations of TRPS I and normal IGF-1 levels. The proband had short stature. Because of her young age, the proband’s parents refused GH treatment. Her older sister had CPP and received 32 months of GNRHa therapy. At the last follow-up, she had reached a normal near-adult height. The older sister’s final height improved to normal levels after GNRHa treatment, which can postpone premature closure of the growth plates [37]. GNRHa therapy for TRPS I patients with CPP has not been reported previously.

## Conclusions

Individuals with syndromic short stature show genetic and phenotypic heterogeneity, making it difficult to obtain a definite diagnosis. This study emphasizes the importance of Trio-WES in patients with syndromic short stature suspected of having a genetic disorder. A definitive molecular diagnosis provides an important basis for prognosis prediction, therapy guidance and genetic counselling. Our study demonstrated that short-term GH treatment had positive effects on hypochondroplasia and PHP-Ia. We advocate studies with larger samples to obtain more evidence of the efficacy of GH treatment in patients with syndromic short stature.

## Data Availability

The datasets generated or analysed during this study are available in the Mendeley repository, https://data.mendeley.com/datasets/3dytgcwgk7/1.

## Abbreviations

CNV: Copy number variant
rhGH: Recombinant human growth hormone
Trio-WES: Trio-whole-exome sequencing
SD: Standard deviation
SNPs: Single nucleotide polymorphisms
MAF: Minor allele frequency
ACMG: American College of Medical Genetics
OMIM: Online Mendelian Inheritance in Man
HGMD: Human Gene Mutation Database
DGV: Database of Genomic Variants
HbA1C: Haemoglobin A1c
INS: Insulin
IGF-1: Insulin-like growth factor-1
PHPV: Persistent hyperplasia of primary vitreous
*FGFR3*: Fibroblast growth factor receptor 3
CA: Chronological age
BA: Bone age
T3: Triiodothyronine
T4: Thyroxine
FT3: Free triiodothyronine
FT4: Free thyroxine
TSH: Thyroid-stimulating hormone
PH: Pubic hair
iPTH: Parathyroid hormone
FSH: Follicle-stimulating hormone
LH: Luteinizing hormone
PRL: Prolactin
P: Progesterone
E2: Oestradiol
T: Testosterone
CPP: Central precocious puberty
GNRHa: Gonadotropin releasing hormone agonist
VUS: Variant of uncertain significance
PHP-Ia, Ib, Ic: Pseudohypoparathyroidism Ia, Ib, and Ic
TRPS I: Trichorhinophalangeal syndrome type I
PPHP: Pseudopseudohypoparathyroidism
POH: Progressive osseous heteroplasia
OC: Osteoma cutis
AHO: Albright’s hereditary osteodystrophy
NESP55: Neuroendocrine secretory protein 55
